# Impact of Breathing and Anatomical Constraints on Subxiphoid Epicardial Puncture: Insights From a Japanese Cohort

**DOI:** 10.1002/joa3.70157

**Published:** 2025-07-30

**Authors:** Natnicha Pongbangli, Hirotsugu Ikewaki, Kyoko Hoshida, Kyoko Soejima

**Affiliations:** ^1^ Department of Cardiovascular Medicine Kyorin University School of Medicine Mitaka Japan; ^2^ Division of Cardiology, Department of Internal Medicine Chiangrai Prachanukroh Hospital Chiang Rai Thailand

**Keywords:** cardiac ultrasound, epicardial access, pericardium distance, respiration, subxiphoid approach

## Abstract

**Background:**

The subxiphoid approach is increasingly utilized for epicardial interventions. Understanding the effect of respiration on the distance from the xiphoid process to the pericardium is essential for improving procedural safety and efficacy.

**Methods:**

A cross‐sectional study was conducted on 51 patients undergoing preprocedural ultrasound imaging. Measurements were taken in the supine position during spontaneous breathing at end‐inspiration and end‐expiration, with the probe directed toward both the midline and the left shoulder. Differences between respiratory phases and probe orientations were analyzed.

**Results:**

The mean xiphoid‐to‐pericardium distance increased significantly from inspiration to expiration for both probe orientations (midline: 42.2 ± 12.2 mm vs. 54.6 ± 14.1 mm, *p* < 0.001; left shoulder: 40.5 ± 12.3 mm vs. 51.2 ± 14.2 mm, *p* < 0.001). The expiration‐inspiration difference was greater with the midline direction (mean difference: 12.4 mm) than toward the left shoulder (10.7 mm). Probe direction affected measurements during expiration (*p* = 0.012) but not during inspiration (*p* = 0.104). The distance to the pericardium showed a positive correlation with body weight (*r* = 0.561), body mass index (*r* = 0.675), and chest dimensions, including anteroposterior (AP) (*r* = 0.477) and lateral diameters (*r* = 0.451). In contrast, the chest wall size index (lateral/AP ratio) was negatively correlated (*r* = −0.365). No significant difference in this distance was found between patients with and without chronic obstructive pulmonary disease.

**Conclusions:**

Respiratory phase and anthropometric parameters significantly influence the distance to the pericardium. These findings may guide safer planning of subxiphoid epicardial procedures.

## Graphical abstract

This study reveals that respiratory phase and anthropometric parameters significantly impact the xiphoid‐to‐pericardium distance, with expiration increasing the depth. These findings support the use of preprocedural ultrasound to optimize subxiphoid epicardial access, enhance safety, and inform needle selection based on individual anatomical characteristics.

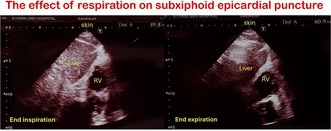

## 1 Introduction

Sub‐xiphoidal percutaneous access to the epicardium is a technique used for pericardiocentesis and electrophysiological interventions, particularly in procedures like epicardial ablation [1]. It involves accessing the epicardial space through a sub‐xiphoidal approach, typically under fluoroscopic or ultrasound guidance [2, 3, 4]. A previous study using magnetic resonance imaging (MRI) to evaluate diaphragmatic motion found that both diaphragmatic and cardiac movements are greater in the supine position compared to the sitting position among healthy volunteers [5, 6]. In the supine position, the strongest correlation was found between cranio‐caudal diaphragmatic movement and cardiac motion [6, 7]. Some studies evaluating diaphragmatic excursions during forced breathing using dynamic chest phrenicography found that higher body mass index (BMI) and vital capacity (VC) were associated with greater diaphragmatic excursions [8]. However, the study was conducted during health screenings with participants in a standing position, which is not the typical position for epicardial puncture.

Dynamic chest radiography is also useful for assessing diaphragmatic function [9], but it is not always readily available and carries the risk of radiation exposure. The assessment of diaphragmatic function and guidance for epicardial access using ultrasound is widely adopted globally and is practical in emergency situations. This method is preferred due to its real‐time visualization capabilities without radiation exposure compared to other imaging techniques [10].

Epicardial puncture via subxiphoid access may be performed during different phases of respiration, reflecting variability in procedural strategies and operator preferences. The SAFER approach recommends performing epicardial puncture at end‐expiration, proposing that sustained apnea in this phase reduces respiratory motion of intra‐abdominal and mediastinal structures, thereby facilitating safer and more precise epicardial access during percutaneous procedures [11]. A previous imaging study supported that breath‐holding at end‐expiration significantly reduces respiratory motion artifacts compared to breath‐holding at end‐inspiration [12]. However, during expiration, when the diaphragm is up [13], the distance to the pericardium increases, and abdominal organs move closer to the xiphoid area, so interaction becomes more likely, especially with the liver or colon [14, 15]. This positional change may elevate the risk of interaction with these organs and potentially increase the risk of iatrogenic injury.

In contrast, the 2025 Expert Consensus Statement allows for flexibility, recommending that the needle be advanced under live fluoroscopic guidance during either an inspiratory breath hold or apnea (if the patient is intubated) [16]. This variability in clinical practice highlights a knowledge gap regarding the optimal respiratory phase for epicardial access. To address this, we conducted the present study to evaluate how respiratory phase affects anatomical distances relevant to epicardial procedures.

The primary objective of this study was to assess the effect of respiration on the distance from the xiphoid process to the pericardium, as measured by ultrasound in different probe orientations. The entry site for epicardial access is typically located lateral to the xiphoid process, just below the margin of the left‐sided rib cage. To simulate real‐world variations in epicardial access techniques, we measured the distance from the subxiphoid entry point to the pericardium in two directions, corresponding to the anterior approach and the posterior approach. The anterior approach generally directs the needle more cranially, toward the chin, avoiding the internal mammary artery and typically traversing less lung tissue and avoiding intra‐abdominal organs such as the liver. In contrast, the posterior approach often directs the needle toward the patient's left shoulder, a trajectory that may carry higher risks due to its closer proximity to the liver and lungs [16]. Our dual‐direction measurement strategy allowed for a comparative evaluation of both used access paths.

A secondary aim was to explore correlations between this distance and patients' anthropometric and chest wall characteristics. By identifying predictable factors that influence pericardial depth, this study seeks to inform more personalized and safer approaches to epicardial access. Ultimately, our findings may contribute to refining procedural planning, equipment selection, and training in epicardial techniques.

## 2 Methods

### 2.1 Study Design and Patient Population

We conducted a cross‐sectional observational study involving 51 consecutive patients scheduled for cardiac ablation procedures at Kyorin University Hospital, Japan. The study was approved by the Institutional Review Board of Kyorin University Hospital (R04‐136). All patients provided consent, and this study did not affect their treatment. All ultrasound assessments were performed in the supine position using a phased array transducer. The distance from the xiphoid process to the pericardium was measured during spontaneous breathing at both end‐inspiration and end‐expiration. The ultrasound probe was positioned just below the xiphoid process and directed toward the midline and left shoulder to obtain a subcostal view. For the anterior approach, a shallow angle of 15°–30° along the midline is typically used, whereas the posterior approach requires a steeper angle of 45°–60° directed toward the left shoulder. We used ultrasound as the principal tool in this study due to its widespread availability in procedure rooms, real‐time visualization capabilities, and ability to avoid unnecessary radiation [17, 18].

Patients were instructed to perform deep inspiration and deep expiration, with measurements taken during brief pauses at each respiratory phase. The pericardium was identified as a thin, echogenic, continuous line surrounding the heart, and the shortest distance from the xiphoid process to this structure was recorded. The targeted measurement point on the epicardium corresponded to the anterior surface of the right ventricle. All measurements were performed by experienced cardiac electrophysiologists, blinded to the study outcomes. Exclusion criteria included known diaphragmatic paralysis, constrictive pericarditis, a diagnosis of pericarditis within the past 3 months, history of open‐heart surgery, coronary artery bypass grafting (CABG), pericardiectomy, prior thoracotomy or lung surgery, and the presence of severe subcostal skin lesions. These conditions may interfere with diaphragmatic motion, cardiac anatomy, or image acquisition.

Baseline patient data were collected, including age, sex, height, weight, comorbidities, and the index procedure. Additional anatomical data were obtained from preprocedural computed tomography (CT) scans, including chest wall diameters and subxiphoid fat thickness. The chest wall size index (CWSI) was calculated as the ratio of lateral to anteroposterior (AP) chest diameters.

### 2.2 Statistical Analysis

Continuous variables were expressed as mean ± standard deviation (SD) for normally distributed data, or as median with interquartile range (IQR) for non‐normally distributed data. Categorical variables were reported as counts and percentages. Paired *t*‐tests were used to compare the xiphoid‐to‐pericardium distances between inspiratory and expiratory phases. Spearman correlation coefficients were calculated to assess associations between anatomical measurements and body size parameters. Univariable and multivariable logistic regression analyses were performed to evaluate factors that might predict hepatic overlapping cases. A *p* < 0.05 was considered statistically significant. Statistical analyses were performed using STATA software version 16.

## 3 Results

The study included 51 patients, of whom 68.6% were male, with a median age of 70 years (interquartile range [IQR] 61–76). The mean height was 163.3 ± 9.0 cm, with a mean body weight of 67.3 ± 13.6 kg and a mean body mass index (BMI) of 25.1 ± 3.7 kg/m^2^. Common comorbidities included hypertension in 70.6% of patients, atrial fibrillation in 82.3%, and heart failure in 27.4%. Other conditions such as diabetes mellitus (23.5%), coronary artery disease (15.7%), chronic obstructive pulmonary disease (11.7%), sleep apnea (7.8%), and prior stroke (3.9%) were also noted. The median left ventricular ejection fraction (LVEF) was 61.6% (IQR 58–64), and the median left atrial size was 38 mm (IQR 35–40), with a left atrial volume index of 35.2 mL/m^2^ (IQR 28.7–42.9). Most patients (80.4%) underwent atrial fibrillation ablation, with 58.8% receiving pulsed field ablation and 35.3% undergoing radiofrequency ablation (Table 1).

**TABLE 1:** Baseline characteristic of patients.

	*N* = 51
Male (%)	35 (68.6)
Mean age (years), IQR	70 (61, 76)
Height (cm), mean ± SD	163.3 ± 9.0
Body weight (kg), mean ± SD	67.3 ± 13.6
BMI (kg/m^2^), mean ± SD	25.1 ± 3.7
Body surface area (m^2^), mean ± SD	1.7 ± 0.2
Comorbid diseases	
Heart failure (%)	14 (27.4)
Hypertension (%)	36 (70.6)
Diabetes (%)	12 (23.5)
Coronary artery disease (%)	8 (15.7)
COPD (%)	6 (11.7)
Sleep apnea (%)	4 (7.8)
History of stroke (%)	2 (3.9)
Atrial fibrillation (%)	42 (82.3)
Echocardiography parameters	
LVEF (%), IQR	61.6 (58, 64)
LA size (mm), IQR	38 (35, 40)
LA volume index, IQR	35.2 (28.7, 42.9)
Index procedure	
AF ablation (%)	41 (80.4)
VT ablation (%)	3 (5.8)
CTI ablation (%)	1 (1.9)
SVT cases (%)	2 (3.7)
PVC cases (%)	2 (3.7)
LAA occlusion (%)	1 (1.9)
Other (%)	1 (1.9)
Ablation source	
Radiofrequency ablation (%)	18 (35.3)
Pulse field ablation (%)	30 (58.8)
Parameters from CT preoperative procedure	
Prone position (%)	32 (68.1)
Distance from xiphoid process to pericardium (mm), IQR	46.8 (41.6, 55.1)
Subxiphoid fat tissue thickness (mm), IQR	20.2 (15.8, 25)
Chest cavity: Anteroposterior diameter (mm), IQR	110 (94.2, 123.9)
Chest cavity: Lateral diameter (mm), IQR	265 (240.3, 281.6)

Abbreviations: AF, atrial fibrillation; BMI, body mass index; COPD, chronic obstructive pulmonary disease; CT, computed tomography; CTI, cavotricuspid isthmus; IQR, interquartile range; LA, left atrium; LAA, left atrial appendage; LVEF, left ventricular ejection fraction; mm, millimeters; PVC, premature ventricular complex; SD, standard deviation; SVT, supraventricular tachycardia; VT, ventricular tachycardia.

The effect of respiration on the distance from the xiphoid process to the pericardium and comparison between probe orientations directed toward the midline (anterior approach) and toward the left shoulder (posterior approach) is shown in Table 2. Ultrasound measurements revealed that the distance from the xiphoid process to the pericardium significantly increased during expiration compared to inspiration, regardless of the probe direction. With the probe directed toward the left shoulder, the mean distance increased from 40.5 ± 12.3 mm at end‐inspiration to 51.2 ± 14.2 mm at end‐expiration (mean difference 10.7 mm; 95% CI: 9.1–12.3; *p* < 0.001). Similarly, when the probe was directed toward the midline, the distance increased from 42.2 ± 12.2 mm to 54.6 ± 14.1 mm (mean difference 12.4 mm; 95% CI: 10.6–14.2; *p* < 0.001). When comparing the two probe orientations, the distance during end‐inspiration did not differ significantly (midline: 42.2 ± 12.2 mm vs. left shoulder: 40.5 ± 12.3 mm; *p* = 0.104). However, during end‐expiration, the midline orientation yielded a significantly longer distance than the left shoulder direction (54.6 ± 14.1 mm vs. 51.2 ± 14.2 mm; mean difference 3.4 mm; 95% CI: 0.7–5.7; *p* = 0.012).

**TABLE 2:** Effect of respiration on the distance from the xiphoid process to the pericardium and comparison between probe orientations directed toward the midline (anterior approach) and toward the left shoulder (posterior approach).

Respiratory phase	Probe direction	Distance mean ± SD (mm)	Mean difference (95% CI) versus inspiration (mm)	Mean difference (95% CI) versus probe direction to left shoulder	*p*
End‐inspiration	Left shoulder	40.5 ± 12.3	—	—	—
	Midline	42.2 ± 12.2	—	1.7 (−0.3–3.1)	0.104
End‐expiration	Left shoulder	51.2 ± 14.2	10.7 (9.1–12.3)	—	< 0.001
	Midline	54.6 ± 14.1	12.4 (10.6–14.2)	—	< 0.001
			—	3.4 (0.7–5.7)	0.012

Abbreviations: CI, confidence interval; mm, millimeters; SD, standard deviation; U/S, ultrasound.

**FIGURE 1:**
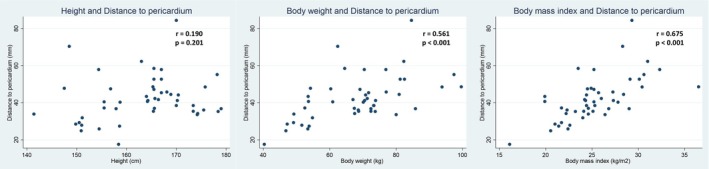
Correlation between body size and the distance to the pericardium. Correlation analyses revealed that height was not significantly associated with the distance (*p* = 0.201). In contrast, body weight (*r* = 0.561, *p* < 0.001) and body mass index (BMI) (*r* = 0.675, *p* < 0.001) all demonstrated strong positive correlations with the measured distance.

**FIGURE 2:**
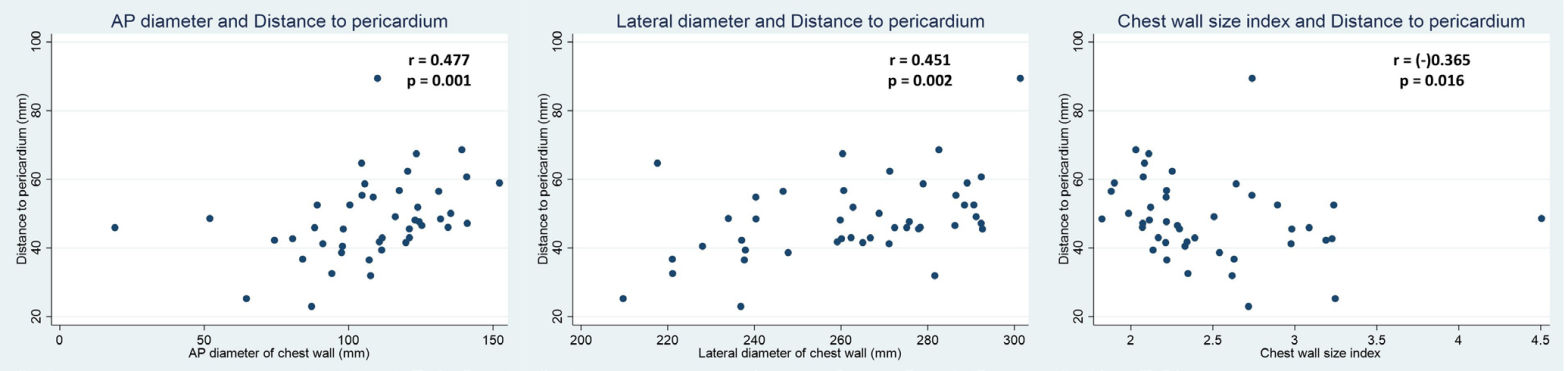
Correlation between chest wall dimensions and the distance to the pericardium. Both anteroposterior (AP) diameter (*r* = 0.477, *p* = 0.001) and lateral diameter (*r* = 0.451, *p* = 0.002) were positively correlated with the measured distance. In contrast, the chest wall size index (CWSI), defined as the ratio of lateral to AP diameter, showed a negative correlation (*r* = −0.365, *p* = 0.016), indicating that a relatively broader chest may be associated with a shallower pericardial location.

**FIGURE 3:**
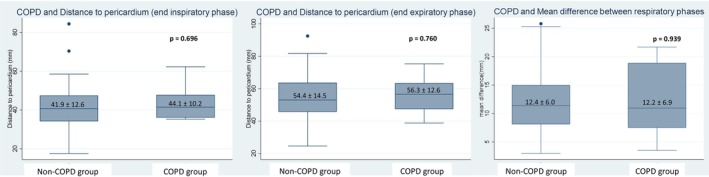
Comparison of the xiphoid‐to‐pericardium distance in patients with and without chronic obstructive pulmonary disease (COPD) shows that there was no significant difference in the xiphoid‐to‐pericardium distance, regardless of the respiratory phase.

Correlation analyses demonstrated that body weight (*r* = 0.561, *p* < 0.001) and body mass index (BMI) (*r* = 0.675, *p* < 0.001) were significantly and positively correlated with the distance from the xiphoid process to the pericardium, whereas height did not show a significant association (*p* = 0.201) (Figure 1). Regarding chest wall dimensions, both anteroposterior (AP) diameter (*r* = 0.477, *p* = 0.001) and lateral diameter (*r* = 0.451, *p* = 0.002) were positively correlated with the distance, while the chest wall size index (CWSI), defined as the ratio of lateral to AP diameter, was negatively correlated (*r* = −0.365, *p* = 0.016), suggesting that a relatively broader chest is associated with a shallower pericardial location (Figure 2). Additionally, there was no significant difference in the xiphoid‐to‐pericardium distance between patients with and without chronic obstructive pulmonary disease (COPD), regardless of the respiratory phase (Figure 3).

**FIGURE 4:**
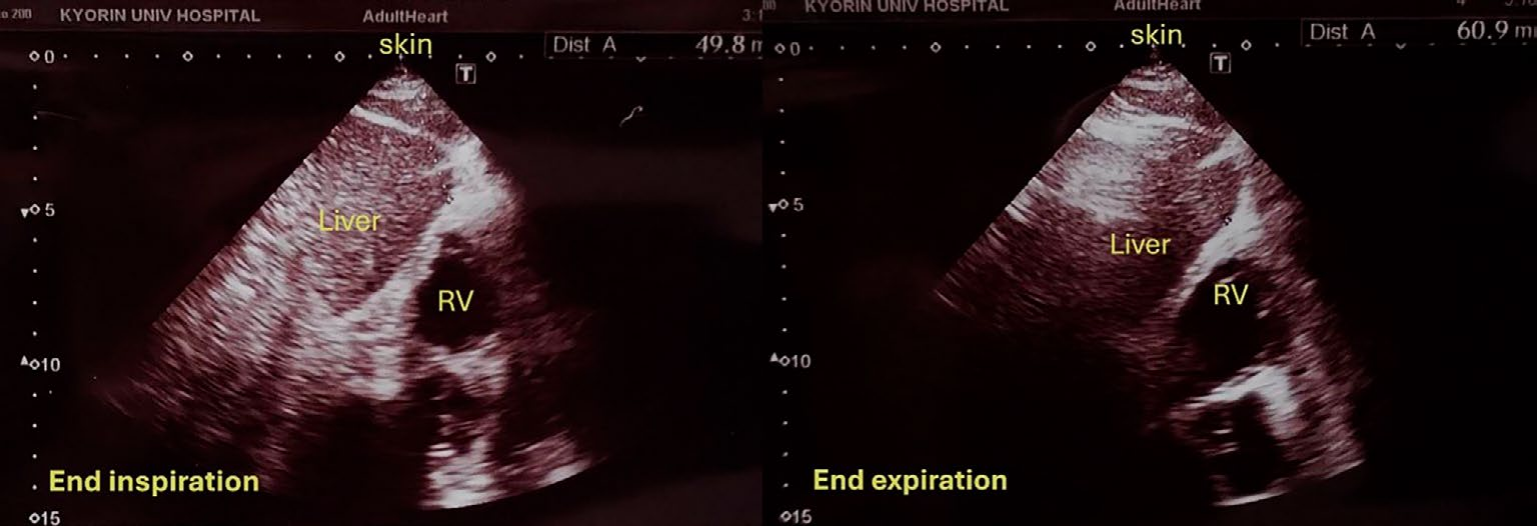
Ultrasound image demonstrating a representative case in which the liver overlaps the subxiphoid trajectory to the epicardial space during both inspiratory and expiratory phases, with more pronounced overlap during expiration. The image was obtained using the anterior (midline‐directed) probe orientation. RV, right ventricle.

**FIGURE 5:**
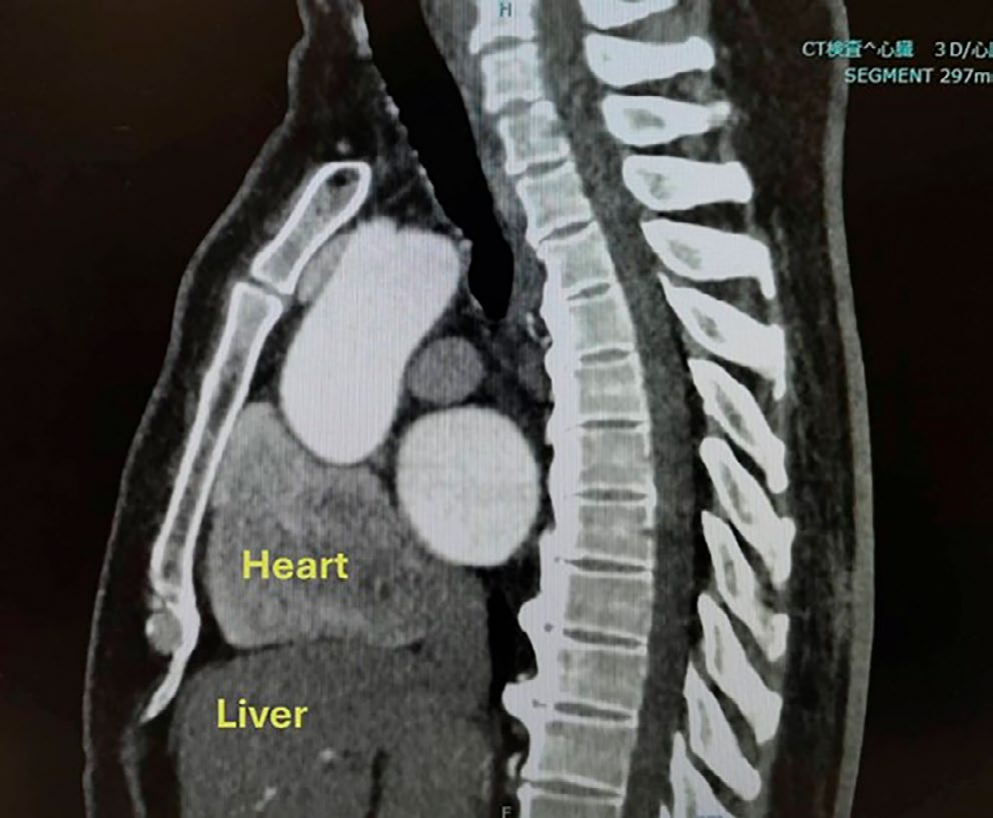
Sagittal computed tomography (CT) image at the subxiphoid level demonstrating a representative case where the liver overlaps the subxiphoid trajectory to the epicardial space.

Among 51 patients, 8 (15.7%) had hepatic overlapping beneath the subxiphoid process. (Figures 4 and 5) In these cases, the liver lies in the direction of puncture, making epicardial access more difficult and potentially hazardous. The remaining 43 patients (84.3%) had no hepatic overlap. There were no significant differences between the groups in terms of age, sex, height, body weight, BMI, or comorbidities such as heart failure, COPD, or atrial fibrillation. Echocardiographic parameters, including LVEF and left atrial volume index, were also similar. Preoperative CT showed that the xiphoid‐to‐pericardium distance was significantly greater in the overlapping group (58.7 ± 16.4 mm) compared to the non‐overlapping group (46.3 ± 9.9 mm; *p* = 0.009) (Table S1). Univariable logistic regression identified a longer xiphoid‐to‐pericardium distance as significantly associated with hepatic overlap (OR 1.09, 95% CI: 1.00–1.19; *p* = 0.031); however, this was not significant in the multivariable model (OR 1.14, 95% CI: 0.92–1.40; *p* = 0.209) (Table S2).

## 4 Discussion

Our study demonstrates that the respiratory phase significantly influences the distance from the xiphoid process to the pericardium, with a notable increase in distance during end‐expiration compared to end‐inspiration. While the clinical consensus statement [16] recommends performing pericardial access either at end inspiration or during apnea, the SAFER epicardial approach [11] specifically utilizes the end‐expiratory phase. The SAFER study demonstrated that sustained apnea at end expiration minimizes respiratory motion of intra‐abdominal and mediastinal structures, thereby facilitating safer and more controlled epicardial access during percutaneous procedures [11] although our study found that the subxiphoid‐to‐pericardium distance may be shorter at end inspiration, epicardial access at this phase can be technically challenging due to abdominal distension. In clinical practice, anterior epicardial access often requires manual abdominal compression, which is more feasible at end expiration when intra‐abdominal pressure is lower [11] Operators who adopt the SAFER technique should note that the distance from the skin to the pericardial space may be greater compared to measurements taken at end inspiration, as recommended in the clinical consensus statement. Moreover, although the subxiphoid‐to‐pericardium distance is shorter at end inspiration, the simultaneous increase in right ventricular size due to enhanced venous return may reduce the pericardium‐to‐RV distance, and operators should be cautious of the potentially increased risk of cardiac injury during access.

The magnitude of change depends on probe orientation. When the operator uses an anterior epicardial approach with the probe directed toward the midline, the distance from end inspiration to end expiration increases by approximately 12 mm. In contrast, with a posterior epicardial approach where the probe is directed toward the patient's left shoulder, the increase is about 10 mm. It may be particularly relevant in patients with borderline anatomical accessibility or when using shorter pericardial access needles. A Tuohy needle has been commonly used, as it features a design that enhances safety and efficacy, minimizing the risk of injuries to cardiac structures or coronary vessels. However, the longest Tuohy needle available in Japan is 8 cm. Although the mean xiphoid‐to‐pericardium distance at end expiration (54.6 ± 14.1 mm) falls within the reach of the maximum available Tuohy needle length, it is worth noting that 2 out of 51 cases (3.9%) had end‐expiratory distances exceeding 80 mm. This highlights a potential procedural limitation in a minority of patients, where standard needle lengths may be insufficient to safely reach the pericardial space. Awareness of this variability is clinically relevant for procedural planning, particularly in patients with larger body habitus.

Furthermore, anthropometric parameters such as body weight, BMI, and chest wall dimensions were positively correlated with the xiphoid to pericardium distance. Larger AP and lateral chest diameters were associated with increased distance. However, a higher chest wall size index (CWSI), defined as the lateral‐to‐AP diameter ratio, was associated with a shorter distance, reflecting a flatter, wider thoracic configuration. In such cases, the heart and pericardium may be positioned closer to the subxiphoid surface, potentially facilitating epicardial access even in individuals with larger body size. These findings suggest that thoracic geometry, not just absolute size, influences procedural feasibility.

Our study also evaluated the impact of intraabdominal organ positioning, particularly the liver, on epicardial access via the subxiphoid route. Liver puncture has been reported in previous studies [19, 20], and in our cohort, 15.7% of patients exhibited hepatic overlap along the projected needle path. These patients had a significantly longer xiphoid‐to‐pericardium distance, suggesting that liver positioning beneath the subxiphoid area may obstruct or complicate ultrasound‐guided access.

This finding is consistent with known liver movement: during expiration, the diaphragm relaxes and moves upward. The liver moves upward and slightly posterior; during inspiration, the diaphragm contracts and moves downward. The liver is pushed downward and slightly forward [14]. Interestingly, hepatic overlap was not reliably predicted by anthropometric variables such as BMI or height, indicating that individual anatomical variation plays a greater role than overall body size. Although xiphoid‐to‐pericardium distance was associated with liver overlap in univariable analysis, it did not remain significant in multivariable models possibly due to the limited number of overlapping cases or collinearity with other factors. While heart failure can cause hepatic congestion and alter liver position, no such association was found in our study, possibly because most patients were clinically stable and decongested before undergoing ablation.

This study offers valuable insights into respiratory phase‐related changes in pericardial depth using real‐time ultrasound, which allows dynamic assessment without radiation exposure during spontaneous breathing. This makes it especially feasible in emergency situations or when general anesthesia is unavailable, which are common limitations in clinical practice. The incorporation of detailed anthropometric and chest wall geometry enables correlation analyses that support individualized procedural planning. Moreover, focusing on patients undergoing catheter ablation enhances the clinical relevance, as epicardial access is commonly considered in these cases.

## 5 Limitations

The study also has several limitations. The sample size was relatively small, and all data were collected at a single center, which may limit the generalizability of the results. Additionally, although measurements were obtained by experienced operators using a standardized protocol, interobserver variability was not assessed. In broader clinical practice, particularly in settings with less ultrasound expertise, greater variability in measurements may occur. Furthermore, while the impact of COPD was examined, other thoracic or abdominal conditions that could influence diaphragmatic position or pericardial dynamics were not evaluated. We also did not assess the presence of bowel gas along the puncture trajectory, as detection is limited by the resolution and reliability of ultrasound. Lastly, although significant anatomical correlations were identified, the study did not assess associations with procedural outcomes such as success rates, needle depth, or complication rates, which remain an important area for future research.

## 6 Conclusions

This study demonstrates that respiration significantly affects the xiphoid‐to‐pericardium distance, with expiration notably increasing the depth regardless of probe direction. Additionally, anthropometric factors and chest wall dimensions further influence this distance. Incorporating preprocedural ultrasound assessment into clinical practice allows for more accurate anticipation of anatomical variability, facilitating optimal needle selection and enhancing the safety and precision of subxiphoid epicardial procedures. Individualized planning based on these parameters may contribute to improved procedural outcomes.

## Ethics Statement

This study was approved by the Institutional Review Board of Kyorin University Hospital (R04‐136) and conducted in accordance with the principles outlined in the Declaration of Helsinki.

## Conflicts of Interest

The authors declare no conflicts of interest.

## Supporting information


**Data S1:** Supporting Information.

**Video S1:** Ultrasound imaging of the subxiphoid anterior approach with the probe oriented toward the midline. The video illustrates dynamic changes in the distance between the xiphoid process and the pericardium throughout the respiratory cycle, highlighting differences during inspiration and expiration.

**Video S2:** Ultrasound imaging of the subxiphoid posterior approach with the probe angled toward the left shoulder. The video shows respiratory‐related variations in the distance from the xiphoid process to the pericardium during both inspiration and expiration.
