# Supplementary material for: Impact of Breathing and Anatomical Constraints on Subxiphoid Epicardial Puncture: Insights From a Japanese Cohort
Source: J Arrhythm. 2025 Jul 30;41(4):e70157. doi: 10.1002/joa3.70157 (PMC12310301; doi:10.1002/joa3.70157)
Supplement: Supplementary file 1 — Data S1: Supporting Information. [file JOA3-41-e70157-s001.docx]

**Supplementary**

**Supplementary Table 1.** Comparison of Patient Characteristics According to Presence of Hepatic Overlap Beneath the Subxiphoid Process

| **Characteristic** | **Liver Overlap**  **(n = 8)** | **No Overlap**  **(n = 43)** | **P value** |
| --- | --- | --- | --- |
| Male (%) | 7(87.5%) | 28(65.1%) | 0.210 |
| Mean age(years), mean ± SD | 66.3 ± 11.8 | 68.5 ± 8.9 | 0.545 |
| Height (cm), mean ± SD | 167.9 ± 9.2 | 162.4 ± 8.8 | 0.116 |
| Body weight(kg), mean ± SD | 71.3 ± 10.8 | 66.6 ± 14.0 | 0.373 |
| BMI, mean ± SD | 25.2 ± 2.9 | 25.1 ± 3.8 | 0.885 |
| **Comorbid diseases**  Heart failure (%)  Hypertension (%)  Diabetes (%)  Coronary artery disease (%)  COPD (%)  Sleep apnea (%)  History of stroke (%)  Atrial fibrillation (%) | 1(12.5%)  5(62.5%)  3(37.5%)  0(0%)  1(12.5%)  1(12.5%)  0(0%)  7(87.5%) | 13(30.2%)  31(72.1%)  9(20.9%)  8(18.6%)  5(11.6%)  3(6.9%)  2(4.6%)  35(81.4%) | 0.302  0.585  0.310  0.184  0.944  0.594  0.534  0.677 |
| **Echocardiography parameters**  LVEF (%), mean ± SD  LA size (mm), mean ± SD  LA volume index, mean ± SD | 64.3 ± 2.6  36.5 ± 2.1  28.8 ± 4.1 | 59.3 ± 9.1  37.7 ± 6.9  38.8 ± 13.1 | 0.137  0.628  0.053 |
| **Parameters from CT preoperative procedure**  Distance from xiphoid process to pericardium (mm), mean ± SD  Subxiphoid fat tissue thickness (mm),  mean ± SD  Chest cavity: Anteroposterior diameter (mm), mean ± SD  Chest cavity: Lateral diameter  (mm), mean ± SD | 58.7 ± 16.4  22.1 ± 5.6  111.4 ± 12.3  267.1 ± 30.5 | 46.3 ± 9.9  20.7 ± 6.4  106.9 ± 26.4  261.8 ± 22.2 | 0.009  0.588  0.666  0.589 |

SD: standard deviation; BMI: body mass index; COPD: chronic obstructive pulmonary disease; LVEF: left ventricular ejection fraction; LA: left atrium; mm: millimeters; CT: computed tomography

**Supplementary Table 2.** Univariable and Multivariable Analyses of Factors Associated with Hepatic Overlap Beneath the Subxiphoid Process

|  | Univariable | | Multivariable | |
| --- | --- | --- | --- | --- |
|  | Odds Ratio  (95% CI) | P value | Odds Ratio  (95% CI) | P value |
| Age | 0.97 (0.90 - 1.05) | 0.538 | 1.86 (0.61 - 5.66) | 0.269 |
| Sex | 3.75 (0.42 - 33.41) | 0.236 | 3.81 (0.19 - 75.52) | 0.379 |
| Height | 1.08 (0.97 - 1.19) | 0.124 | 0.68 (0.02 - 17.23) | 0.820 |
| Body weight | 1.02 (0.97 - 1.08) | 0.368 | 4.89 (0.49 - 48.60) | 0.175 |
| BMI | 1.01 (0.83 - 1.24) | 0.883 | 0.81 (0.42 - 1.55) | 0.521 |
| Heart failure | 0.33 (0.03 - 2.95) | 0.322 | 1.14 (0.04 - 28.98) | 0.935 |
| COPD | 1.08 (0.11 - 10.75) | 0.944 | 1.21 (0.03 - 50.78) | 0.917 |
| LVEF | 1.11 (0.97 - 1.27) | 0.134 | 3.82 (0.62 - 23.34) | 0.146 |
| LA volume index | 0.90 (0.81 - 1.00) | 0.058 | 0.89 (0.76 - 1.05) | 0.186 |
| Distance from xiphoid process to pericardium | 1.09 (1.00 - 1.19) | 0.031 | 1.14 (0.92 - 1.40) | 0.209 |
| Subxiphoid fat tissue thickness | 1.04 (0.91 - 1.17) | 0.580 | 0.97 (0.77 - 1.22) | 0.796 |
| Chest cavity: Anteroposterior diameter | 1.00 (0.97 - 1.04) | 0.659 | 1.02 (0.95 - 1.10) | 0.488 |
| Chest cavity: Lateral diameter | 1.01 (0.97 - 1.05) | 0.582 | 1.04 (0.94- 1.14) | 0.424 |

BMI: body mass index; COPD: chronic obstructive pulmonary disease; LVEF: left ventricular ejection fraction; LA: left atrium


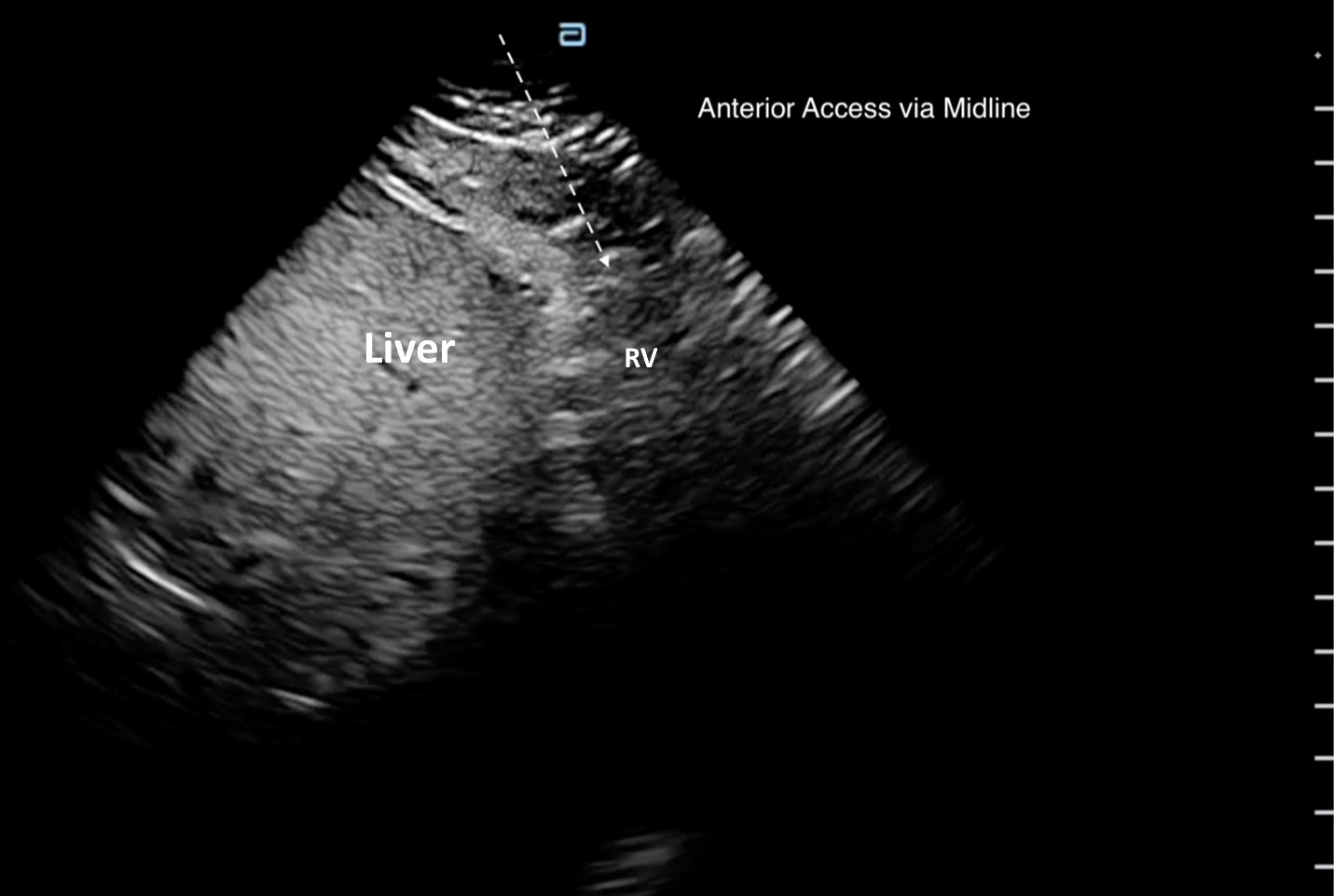


**Supplementary Figure 1.** Representative ultrasound image of the anterior subxiphoid approach, in which the probe is directed toward the patient’s midline. RV: right ventricle


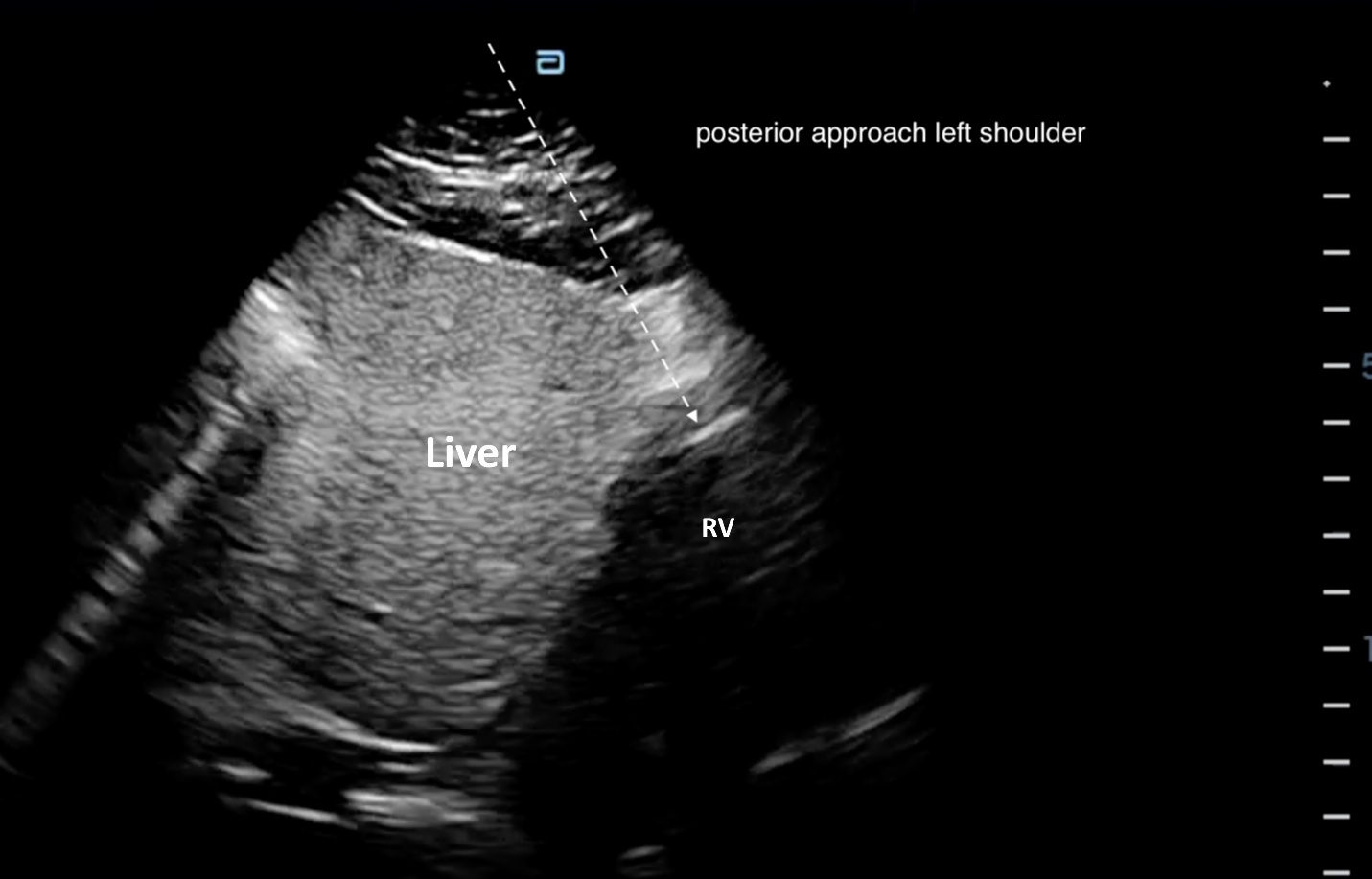


**Supplementary Figure 2.** Representative ultrasound image of the posterior subxiphoid approach, in which the probe is directed toward the patient’s left shoulder. RV: right ventricle
